# Contamination of a drug consumption room with drugs and potential risks for social health care workers

**DOI:** 10.1186/s12954-024-01074-y

**Published:** 2024-08-16

**Authors:** Flore Cuffaro, Georges Dahm, Claude Marson, Patrick Berlemont, Michel Yegles, Claudia Allar, Lionel Fauchet, Matteo Creta, Serge Schneider

**Affiliations:** 1 Laboratoire national de santé, 1, rue Louis Rech, Dudelange, L-3555 Luxembourg; 2 Abrigado, 8, Route de Thionville, L-2610 Luxembourg

**Keywords:** Drug consumption room, Occupational health, Surface contamination, Drugs of abuse, Heroin, Cocaine

## Abstract

**Background:**

Studies have shown that contamination of surfaces by illicit drugs frequently occurs in forensic laboratories when manipulating seized samples as well as in pharmacies and hospitals when preparing medicinal drugs. In this project, we extended these studies to a Drug Consumption Room to investigate drug levels and possible exposure of the staff members.

**Methods:**

We investigated pre and post cleaning contamination by heroin and cocaine and their degradation products 6-monoacetylmorphine and benzoylecgonine on different surfaces (tables, counters, computers and door handles) and in the ambient air. We also collected urine and hair samples from staff members to check for potential short and long term contaminations.

**Results:**

Medium to heavy contamination has been detected on most surfaces and door handles; as expected, air contamination was particularly high in the smoking room. Drug levels were < LOD to very low in the urine and the hair samples of staff members tested.

**Conclusion:**

The cleaning efficiency of the surfaces, carried out by staff and drug users after drug consumption, was often not satisfactory. The very low drug levels in hair indicate that acute health risks for staff members are low.

## Introduction

With the goal of offering a clean and secure environment and reducing acute health risks for hard drug users, low threshold “Drug Consumption Rooms” (DCRs), have been established in many countries since the beginning of the 1990s [1].

At the DCR in Luxembourg City (“Abrigado”), a mean of 141 drug consumptions took place per day in 2022 [2]. The Abrigado is open every day of the week and offers sleeping possibilities, medical support and social counselling for the drug users. A smoking room with 6 and an injection room with 8 places are available. Each of these places is used several times a day. According to Abrigado’s internal statistics, drug injection and drug smoking processes are equally distributed and the heroin/cocaine consumption ratio is estimated at 51% heroin, 28% cocaine and 21% heroin/cocaine mixtures (“speedballs”). Currently, 54 people are working at the Abrigado in the administration, social and medical healthcare or as security and cleaning staff.

Previous studies have shown that surfaces in forensic laboratories [3, 4] and in police stations handling, analyzing seized product [5] may be contaminated by drugs. All major drugs (cocaine, heroin, methamphetamine and fentanyls) have been found on most of the surfaces in a forensic laboratory. Not surprisingly, only background levels of drugs were detected at benches, the report writing sections and significantly higher levels were detected at surfaces used for unpacking and analysis of the samples.

Sisco and Najarro reported that heroin and cocaine were present on 75 and 82% of collected samples respectively [3], also on surfaces generally touched without protection equipment such as doors handles and telephones. The concentration ranges spread over several orders of magnitude (0.002-412 ng/cm^2^ median 2.0 ng/cm^2^ for cocaine and 0.02–456 ng/cm^2^, median 3.2 ng/cm^2^ for heroin) depending of the laboratory section where the samples were collected. Even if no direct health risks were associated with these findings, they may result in safety recommendations for the personal and/or procedure updates for the cleaning staff. A similar study investigating drug contamination over 6 years in hospital pharmacies found highest contamination at locations where the medicines were prepared [6].

As for surface contamination, reports on the contamination of ambient air by drugs are sparse. In 2007 Cecinato and co-workers detected cocaine in the air in 9 out of 11 cities investigated. The concentrations ranged from pg/m^3^ to ng/m^3^. Overall, “the atmospheric concentrations of cocaine correlated to the prevalence of the drug in the Italian regions investigated” [7] and indoor concentrations were generally higher than outdoor concentrations [8]. Some concentrations were > 100 pg/m^3^, exceeding levels of polychlorinated dioxins in the environment [9]. Another study showed that cocaine might be present in the air directly by smoking and indirectly by the transport *via* the cloths or the hair of people in contact with this substance [10]. Particles resulting from drug smoking may persist for several weeks and may convert to other potentially dangerous compounds [11].

To the best of our knowledge, no studies investigating drug contamination of surfaces and of the air have been carried out in DCRs. All staff members of the Abrigado may be in contact with drug users during a normal working day at all premises of the DCR. They are potentially exposed to contaminated surfaces and/or contaminated air.

The major contamination sources are:

- Direct contact with the drugs during the check-in of the drug consumers to the consumption and smoking rooms;
- Contact with contaminated surfaces;
- Exposure to the vapors of the smoked drugs.

No specific cleaning procedures were defined at the Abrigado at the moment of the study; standard commercial desinfectants were used to clean surfaces contaminated with blood, the smoking room was cleaned with soap and hot water.

In this project, we investigated the contamination of 13 surfaces and of the air at 5 spots within the Abrigado. We also tested urine and hair samples from 20 volunteer staff members. The drugs included in the study were heroin (HER), its metabolite 6-monoacteylmorphine (6-MAM), cocaine (COC) and its metabolite benzoylecgonine (BZE). The goals of the project were to assess the levels of air and surface contamination, to identify high risk zones and to quantify potential occupational exposure of the DCR’s staff.

## Materials and methods

### Collection of surface samples

**Fig. 1:**
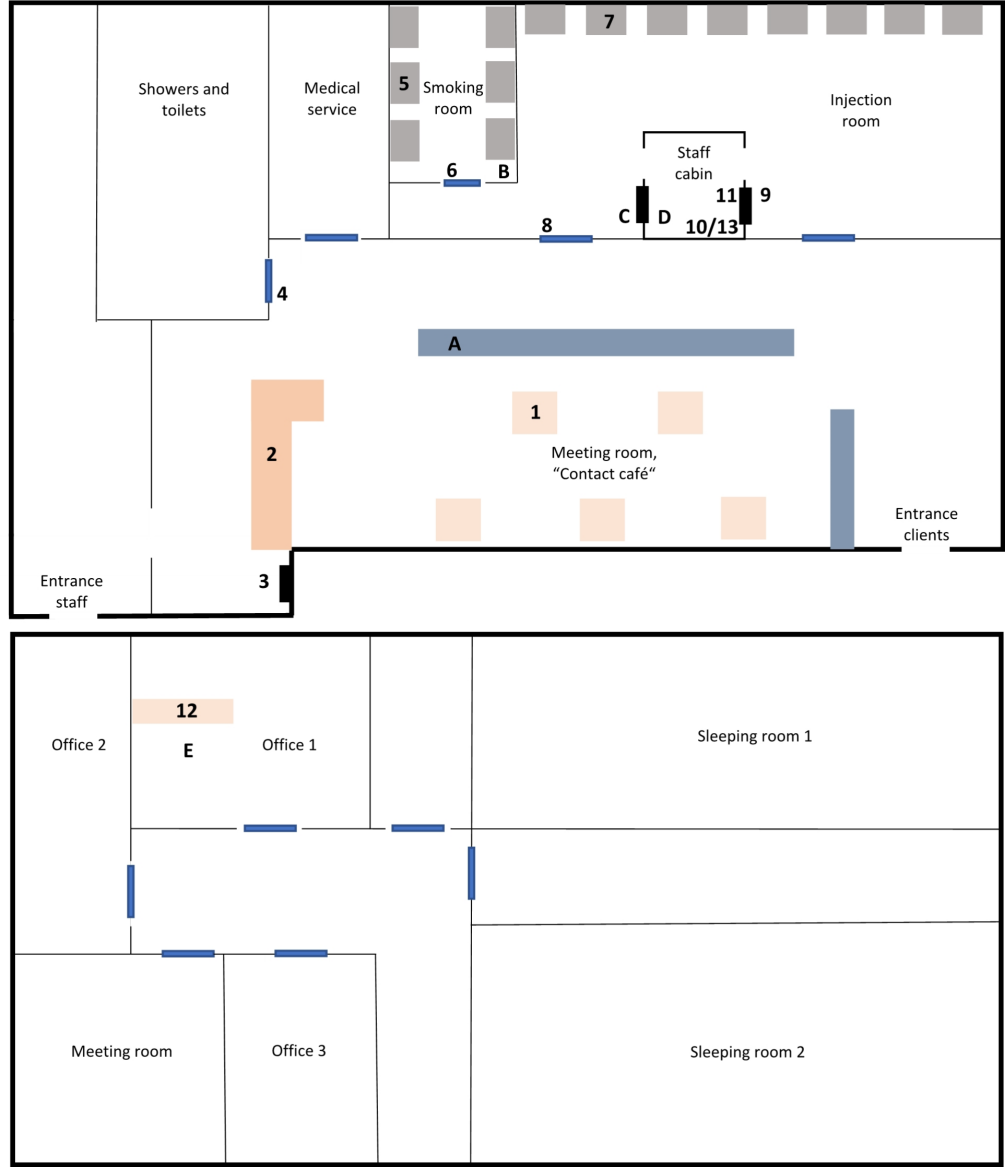
Plan of the ground and first floor of the Abrigado drug consumption room. Surface samples were collected on spots 1 to 13; air samples were collected on spots **A**-**D**

A simplified map of the DCR is presented in Fig. 1. The DCR includes:

- A meeting room for drug users (151.2 m^2^), called “Contact café”, where social interaction occurs, food and drinks may consumed but drug consumption is strictly forbidden. Sampling spots 1 (table), 2 (counter), 3 (window sill for needle exchange) and 4 (door handle) were chosen in the meeting room;
- A smoking room (9.6 m^2^, 30 m^3^) where smoking and inhalation of drugs takes place. Sampling spots 5 (table) and 6 (door handle) were chosen. The smoking room is equipped with an air extraction system (DVEC 200 A roof fan, 0.18 kW, air flow 600 m³/h) active during the opening hours, assuring air replacement every 3 minutes;
- An injection room (60.5 m^2^) where drugs are consumed by injection. Sampling spots 7 (table), 8 (door handle) and 9 (client side counter) were selected;
- A staff cabin (5.0 m^2^) used for handing over clean utensils and verifying the client’s identity before allowing access to the smoking and injection rooms. Sampling spots 10 and 13 (computer keyboard and mouse) and 11 (staff side counter) were selected;
- 3 offices, a meeting room and 2 sleeping rooms are located on the first floor. Sampling spot 12 was chosen on the table of the office room 1.

The staff cabin in the injection room and the meeting room “Contact café” are equipped with respectively one or two air-cleaners (Camfil City-M).

Surface samples were collected each day during 4 days at the 13 spots. Each day “morning samples” were taken after cleaning and before opening of the DCR for the drug users and “evening samples” were taken after closing of the DCR and before cleaning of the rooms. The hypothesis was that the “morning samples” would be clean and that the “evening samples” would be contaminated. Samples from door handles (4, 6 and 8) were taken on both sides of the door and treated as one sample. A total of 104 surface samples were collected using disposable TX714K Large Alpha polyester swabs (Texwipe). The swabs were moistened with methanol (MeOH) before the sample collection. The plane sampling surfaces (tables and counters) had an area of ​​300 cm^2^ (3 times 10 × 10 cm^2^ with disposable cardboard frames). Each 10 × 10 cm^2^ surface was collected twice using both sides of an individual swab and the three swaps were combined. For the handles, keyboard and the computer mouse, the entire object was sampled using a single swab. The swabs were placed in a 25 mL amber bottle and stored in a desiccator with silica gel until analysis in order to prevent degradation.

### Collection of air samples

Active air samples were collected simultaneously at 5 spots (A - E in Fig. 1) for 4 days during the opening hours of the DCR.

The sampling pumps (Gilian-GilAir Plus) were calibrated before and after each use, using the Gilian Gilibrator 3 Calibrator, to generate a flow rate of 2000 mL/min. IOM sampling cassettes were connected to each pump and loaded with 24 mm glass microfiber filters (VWR, 516–0881). Filters were placed at a high of 2 m from the floor. Air was sampled for a mean of 407 min per day; a total of 20 samples were collected. After sampling, the filters were placed in a 25 mL amber bottle and stored in a desiccator until analysis.

### Analysis of surface and air samples

Surface and air samples were analyzed using LC/Q-ToF in the positive ion mode. The method has been described elsewhere [12] and was adapted for HER, 6-MAM, COC and BZE analysis. The calibration linearity was r^2^ > 0.99, the limit of detection (LOD) was 0.06 ng/cm^2^ for surface samples and 3.12 ng/m^3^ for air samples. The limit of quantification (LOQ) was 0.20 ng/cm^2^ for surface samples and 10 ng/m^3^ for air samples for all substances.

Surface swabs were extracted with 15 mL MeOH, vortexed for 30 s at 3000 rpm and then placed in an ultrasonic bath for 15 min. The filters were extracted with 2 mL of MeOH and also placed in an ultrasonic bath for 15 min. Deuterated internal standards (6-MAM-D_3_; BZE-D_3_; Cocaïne-D_3_; Heroïne-D_3_) were added to obtain a final concentration of 350 ng/L. The extraction solutions were diluted with LC eluent before LC-Q-ToF analysis. Dilution factors varied according to surfaces tested and, when necessary, were adapted to fit into the calibration curve. HER, 6-MAM, COC, BZE and their deuterated analogues were purchased from LGC (Molsheim, France).

### Collection of hair and urine samples

Participation was on voluntary basis and 20 (37%) out of the 54 employees of the Abrigado participated. No participant has or had a known history of heroin or cocaine consumption. All participants received a medical prescription for hair and urine analysis and they all provided written consent for anonymized publication of the results. No individual results were communicated to the Abrigado facility management.

The collection of samples took place at the Abrigado. Hair samples were collected from the vertex posterior of the head [13]. The proximal 3 cm segment was used for analysis. The samples were stored at room temperature in aluminium foil before analysis. Urine samples were collected at the end of the working day and stored at 5 °C before analysis.

### Analysis of hair and urine samples

Urine samples (100 µL) were extracted with 1 mL acetonitrile to precipitate residual protein material. The mixture was then centrifuged at 10,000 rpm for 15 min. The supernatant was evaporated and the residues were reconstituted in the LC-MS/MS mobile phase. A targeted screening method for MOR, 6-MAM, COC and BZE was performed on a 6500QTRAP LC-MS/MS (Sciex) using multiple reaction monitoring (MRM). LOD for MOR, 6-MAM, COC and BZE were 0.11, 0.40, 0.01 and 0.04 ng/mL, respectively.

Hair locks were decontaminated twice with methanol, then pulverized in a ball mill and the powered hair was incubated in a phosphate buffer for 2 h in an ultrasonic bath after addition of deuterated internal standards. After solid-phase extraction and derivatization with MSTFA (N-Methyl-N-(trimethylsilyl)trifluoroacetamide, (Marchery-Nagel, Hoerdt, France), the samples were analyzed by GC-MS/MS (Agilent) in EI mode using MRM [14].

The 2 washing solutions used for the hair decontamination were analyzed to investigate passive contamination of the participants. The washing solutions were combined, spiked with the internal standard and after solid phase extraction and derivatization using MSTFA, the analytes COC, BZE, 6-MAM and HER were also determined by GC-MS/MS in EI mode using MRM.

The LOQ for COC, BZE, 6-MAM and HER were respectively 0.36 ng/mg, 0.13 ng/mg. 0.38 ng/mg and 2.82 ng/mg hair.

## Results

### Surface contamination

Out of the single 416 measurements (13 spots, 4 compounds, 4 days, morning and evening collection), all were positive (> LOD) for one or several of the monitored drugs. The most frequently detected compound was COC (100% of all measurements); HER, 6-MAM and BZE were all detected in about two thirds of the measurements. An overview of the results is given in Table 1.

**Table 1:** Detection of COC, HER, 6-MAM and BZE on surfaces

Result	COC	HER	6-MAM	BZE
Not detected (< LOD)	0 (0.0%)	35 (33.7%)	34 (32.7%)	37 (35.6%)
Detected (> LOD)	104 (100%)	69 (66.3%)	70 (67.3%)	67 (64.4%)

Cocaine was not only the most frequently detected compound but also the compound with the highest concentrations measured.

The maximum COC concentration was 1544 ng/cm^2^ (evening sample of the counter of the injection room, sampling spot 9). The maximum BZE concentration was 222 ng/cm^2^ (morning sample of the door handle at the smoking room, sampling spot 6). Maximum HER and 6-MAM concentration levels all were below 60 ng/cm^2^. Median values were near zero for all compounds except COC (15 ng/cm^2^), with a small difference between the morning (9.7 ng/cm^2^) and the evening (33 ng/cm^2^) samples. A summary of the maximum concentrations measured for the 4 compounds is given in Table 2.

**Table 2:** Maximum concentrations levels for COC, BZE, HER and 6-MAM in morning and evening samples

Sample	Maximum concentration measured (ng/cm^2^)	Sampling spot
COC morning	714	door handle smoking room, spot 6
COC evening	1544	counter client side injection room, spot 9
BZE morning	222	door handle smoking room, spot 6
BZE evening	184	door handle smoking room, spot 6
HER morning	4	counter client side injection room, spot 9
HER evening	55	table injection room, spot 7
6-MAM morning	42	door handle smoking room, spot 6
6-MAM evening	54	door handle smoking room, spot 6

The mean surface contamination (ng/cm^2^) for spots 1 to 13 was defined as the mean of the sum of COC, BZE, HER and 6-MAM after the 4 days of sample collection.

**Fig. 2:**
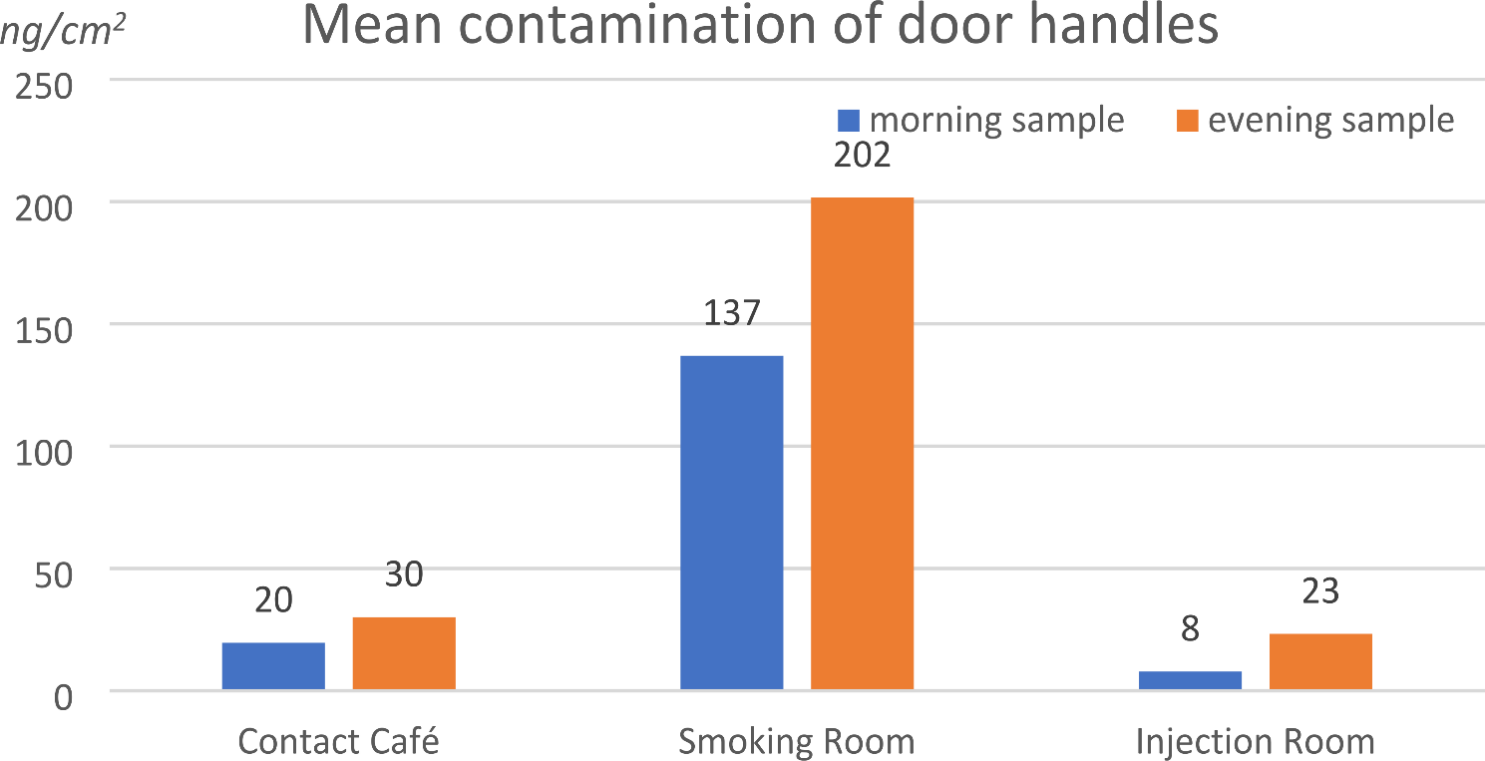
Mean contamination levels (ng/cm^2^) of door handles in the morning after cleaning and in the evening before cleaning

The highest mean surface contamination was measured on the door handle linking the smoking room to the injection room (spot 6). The contamination of the doors handle linking the contact café to the injection room (spot 8) and the contact café to the toilets (spot 4) were of similar magnitude (23 and 30 ng/cm^2^) and were almost ten times lower than mean contamination on spot 6. Contamination was 1.5 to 3 times lower in the morning, after cleaning, than in the evening before cleaning. The mean results for the door handles are presented in Fig. 2.

**Fig. 3:**
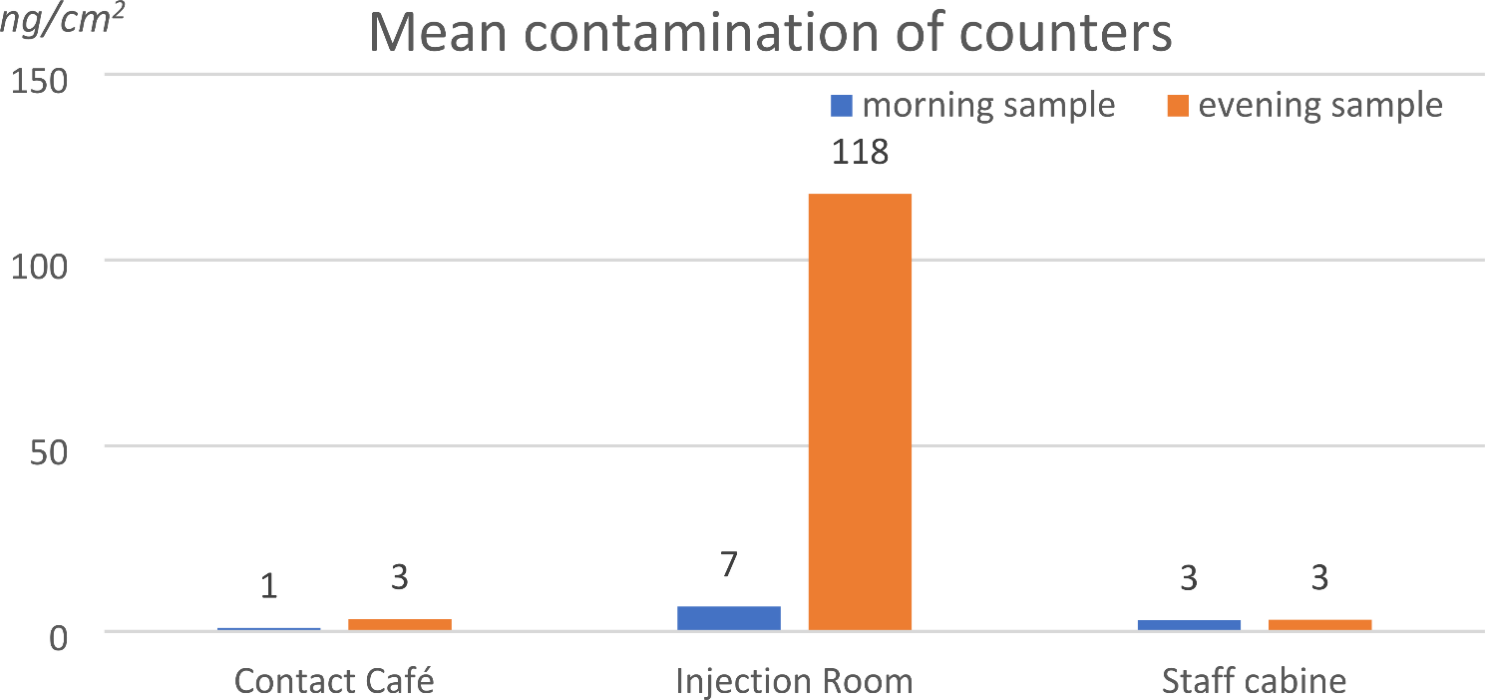
Mean contamination levels (ng/cm^2^) of counters in the morning after cleaning and in the evening

High mean contamination (118 ng/cm^2^) was found at the counter in the injection room (spot 9) in the evening samples. Contamination levels of the counters at the contact café (spot 2) and in the staff cabin (spot 11) were significantly lower (3 ng/cm^2^ for morning and evening samples). Cleaning measures had an obvious effect in the injection room but were never totally efficient. The mean results for the 3 counters are presented in Fig. 3.

**Fig. 4:**
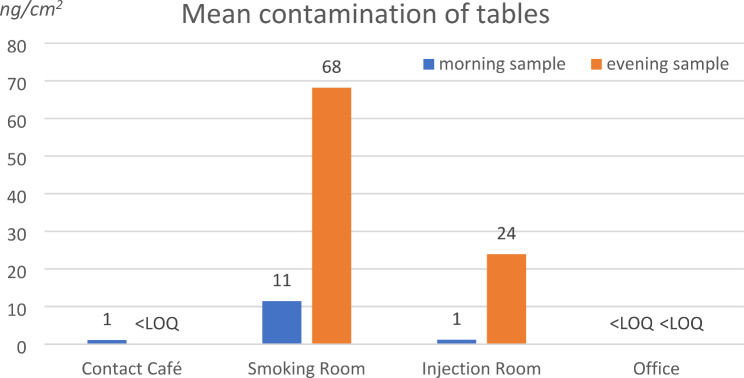
Contamination of tables (ng/cm^2^) after cleaning and after drug use

High mean surface contamination levels were measured in the evening samples on the tables from the smoking (spot 5, 68 ng/cm^2^) and injection rooms (spot 7, 24 ng/cm^2^). Only low contaminations were measured at the table of the contact café (spot 2), which is consistent with the prohibition of drug consumption in this room. Not surprisingly, the lowest contamination levels (< LOQ) were measured at the table in the office room (spot 12). In all cases much lower contamination levels were measured in the morning samples following cleaning. The mean results for the tables are presented in Fig. 4.

Other surfaces analyzed were the window sill for needle exchange (spot 3), the computer in the staff cabin (spot 10) and the bureau at the office on the first floor (spot 12). The mean contamination concentrations were all below the LOQ.

**Fig. 5:**
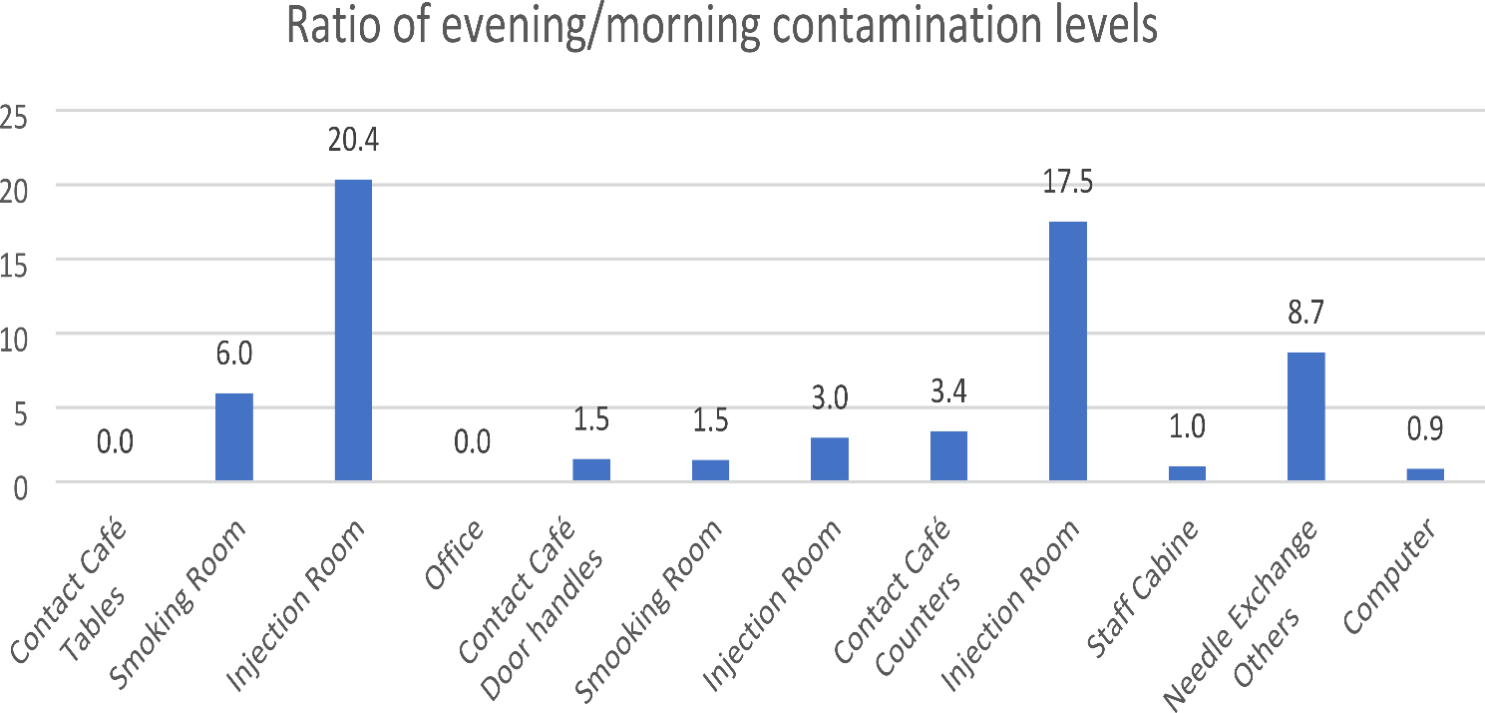
Estimation of the cleaning efficiency (ratio of mean evening/ mean morning contamination levels) for tables, door handles and counters at the DCR

The cleaning efficiency was estimated by calculating the ratio of surface contamination in the evening and surface contamination in the morning, a higher value representing a higher cleaning efficiency. This ratio was highly variable ranging from 0 (no variation) to 17.5 and 20.4 for the counter and table respectively in the in the injection room (Fig. 5).

### Air contamination

Not surprisingly, contamination was by far highest in the smoking room (mean COC: 41.5 µg/m^3^, mean HER + 6-MAM: 41.4 µg/m^3^). The injection room and staff cabin had similar contamination levels for all compounds, indicating passive exposure to smoke of staff members working at this spot. The doors and the counter windows were most often kept open during the opening hours of the DCR, thus allowing constant air exchange between the two rooms.

**Fig. 6:**
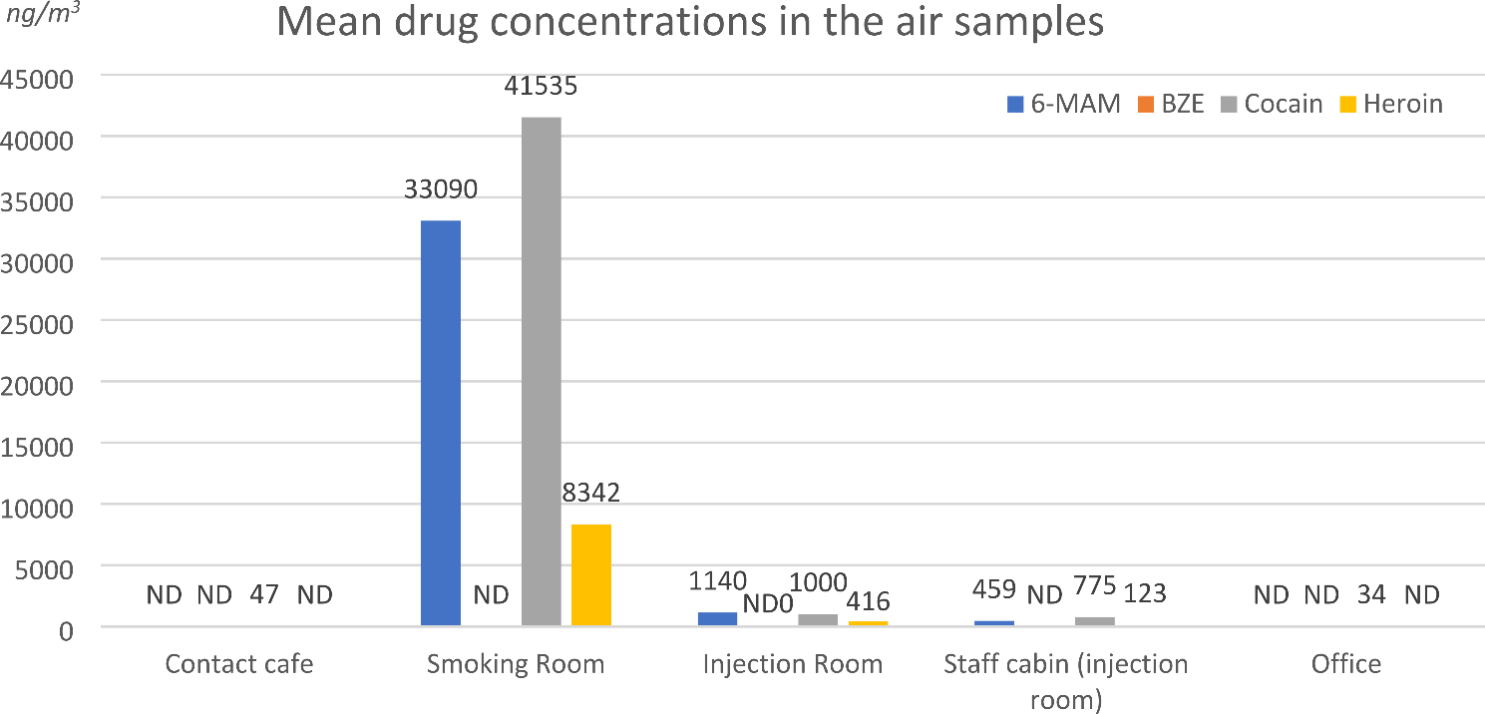
Mean drug concentrations (ng/m^3^) in the air in different locations of the DCR. ND = not detected

Lower levels of cocaine were detected in the contact café and in the office at the first floor. No BZE was detected in any of the air samples, presumably because BZE is not a volatile degradation product of cocaine. The mean concentrations of COC, BZE, HER and 6-MAM obtained after 4 days of sample collection are summarized in Fig. 6.

Considering a mean breathing rate of 12 air breaths/minute and approximately 500 mL of air exchange per breath, approximately 360 L of air are ventilated each hour by a healthy person [15]. Depending on the location within the DCR, this corresponds to passive ingestion ranging over several orders of magnitude from approximately 12 ng to 15 µg of drug and drug metabolites every hour:

- 17 ng/h of COC in the contact café,
- 15,000 ng/h of COC and 14,900 ng/h of HER + 6-MAM in the smoking room,
- 360 ng/h of COC and 560 ng/h of HER + 6-MAM in the injection room,
- 165 ng/h of COC and 209 ng/h of HER + 6-MAM ingested in the staff cabin and.
- 12 ng/h of COC in the office room.

Studies of passive inhalation by volunteers, with a history of cocaine use, inhaling an estimated 250 µg of cocaine (about 16 times above the highest mean concentration level measured at the DCR) resulted in the absence of any pharmacological effects and also in negative urine tests [16].

### Urine and hair samples

All urine samples were below LOD for MOR, 6-MAM, COC and BZE using LC-MS/MS analysis. The exposure at the Abrigado to cocaine and heroin and their metabolites was too low to generate a positive urine result.

The hair washing solutions and the hair extracts were analyzed separately. The washing solutions are an indicator for passive contamination by drug in the ambient air and the extracts are considered as an indicator of passive or active drug ingestion [17].

In the washing solutions:

- COC range was < LOD – 18.8 pg/mg, detected in 19 samples (95%);
- BZE range was < LOD – 1.4 pg/mg, detected in 18 samples (90%);
- HER was < LOD in all 20 samples;
- 6-MAM range was < LOD – 1.7 pg/mg, detected in 2 samples (10%).

In the hair samples:

- COC range was < LOD – 18.3 pg/mg, detected in the hair of 11 participants (55%);
- BZE range was < LOQ – 5.6 pg/mg, the same 11 participants had a positive BZE result;
- HER and 6-MAM were < LOD in all the hair extracts.

The presence of COC and BZE in hair and hair washing solutions and the presence of 6-MAM in washing solutions show that employees may be contaminated by drugs present at the Abrigado, predominately COC. But as concentrations were always far below recommended cut-off values [18] (0.5 ng/mg of hair for cocaine and 0.2 ng/mg of hair for 6-MAM), our results strongly suggest that the presence of drugs results from passive contamination only. A summary of the results is given in Table 3.

**Table 3:** Summary of results (mean, minimum, maximum and median) for hair and hair washing solution of 20 persons working at the DCR

	HAIR				WASHING SOLUTION			
(pg/mg)	COC	BZE	HER	6-MAM	COC	BZE	HER	6-MAM
MEAN	5.1	1.3	< LOD	< LOD	5.9	0.5	< LOD	0.3
MIN	< LOD	< LOD	< LOD	< LOD	< LOD	< LOD	< LOD	< LOD
MAX	18.3	5.6	< LOD	< LOD	18.8	1.4	< LOD	1.7
MEDIAN	3.6	1.1	< LOD	< LOD	4.2	0.4	< LOD	< LOD

## Discussion, recommendations and conclusion

This project is the first aiming to evaluate presence of drugs at surfaces and in the air in a DCR and to evaluate the exposure and potential contamination of staff *via* urine and hair analysis. The substances included in the project were those consumed at the DCR: cocaine, heroin and their degradation products, benzoylecgonine and 6-monoacetylmorphine. The samples were collected over 4 days, in the morning before opening and at the end of the day before closing of the DCR.

Not surprisingly, the mean air contamination was by far highest in the smoking room with > 41 µg/m^3^ for COC and also for HER + 6-MAM. Three out of the 54 staff members had complained at least once about symptoms (headache, nausea and a scratchy throat) when working in this room. Even if these symptoms could not be directly linked to the presence of drugs in the air or the staff’s sporadic presence in the smoking room, short term high drug aerosol levels may influence the wellbeing of the staff members in an acute way and in the long term. Also, no difference in air contamination was observed between the injection room and the staff cabin, presumably because doors between these spaces were left open most of the time.

Door handles were the most contaminated surfaces with levels ≈ 200 ng/cm^2^. This may be important considering that the same doors are used by drug users and DCR staff without any protection i.e. gloves.

Regarding the tables, contamination levels were lower than for door handles but still high in the smoking and the drug injection rooms in the evening before cleaning. Even if the drug users are supposed to clean the tables after usage, this process was obviously not efficient most of the time. Only the counter in the injection room presented a concentration level about 100 ng/cm^2^_,_ as consumers were requested to present their drugs here to the staff.

Despite frequent presence in drug consumption rooms, urine samples of staff members were negative for the drugs investigated. The presence of COC and BZE in hair and hair washing solutions and the presence of 6-MAM in washing solutions however show that employees may be contaminated by drugs present in these places. But, as drug concentrations were largely below the cut-off values, these findings are compatible with passive contamination of hair and not with active drug use. Most important, all drug levels measured in hair and urine samples indicate that no short term health risk was present for the staff members working at the DCR. A long term health risk due to chronic exposure [19] should not be excluded and requires further investigations.

Finally, for preventive and efficient protection against staff contamination, we recommend.

- keeping doors closed, especially the door giving access to the smoking room;
- installation of contactless door opening systems for all doors of the DCR;
- wearing gloves when working at places with potentially high surface contamination;
- assuring an efficient air extraction system and eventually.
- wearing efficient dusk masks (i.e. FFP2) when working in the smoking room.

## Data Availability

No datasets were generated or analysed during the current study.
